# Symmetry-breaking synthesis of Janus Au/CeO_2_ nanostructures for visible-light nitrogen photofixation†

**DOI:** 10.1039/d2sc03863c

**Published:** 2022-10-24

**Authors:** Henglei Jia, Mengxuan Zhao, Aoxuan Du, Yanrong Dou, Chun-yang Zhang

**Affiliations:** College of Chemistry, Chemical Engineering and Materials Science, Shandong Normal University Jinan 250014 China cyzhang@sdnu.edu.cn

## Abstract

Precise manipulation of the reactive site spatial distribution in plasmonic metal/semiconductor photocatalysts is crucial to their photocatalytic performance, but the construction of Janus nanostructures through symmetry-breaking synthesis remains a significant challenge. Here we demonstrate a synthetic strategy for the selective growth of a CeO_2_ semi-shell on Au nanospheres (NSs) to fabricate Janus Au NS/CeO_2_ nanostructures with the assistance of a SiO_2_ hard template and autoredox reaction between Ag^+^ ions and a ceria precursor. The obtained Janus nanostructures possess a spatially separated architecture and exhibit excellent photocatalytic performance toward N_2_ photofixation under visible-light illumination. In this scenario, N_2_ molecules are reduced by hot electrons on the CeO_2_ semi-shell, while hole scavengers are consumed by hot holes on the exposed Au NS surface, greatly promoting the charge carrier separation. Moreover, the exposed Au NS surface in the Janus structures offers an additional opportunity for the fabrication of ternary Janus noble metal/Au NS/CeO_2_ nanostructures. This work highlights the genuine superiority of the spatially separated nanoarchitectures in the photocatalytic reaction, offering instructive guidance for the design and construction of novel plasmonic photocatalysts.

## Abstract 2

We demonstrate a synthetic strategy to selectively grow a CeO_2_ semi-shell on Au nanospheres through the symmetry-breaking synthesis. The asymmetric nanostructures facilitate the charge carrier separation during the visible-light N_2_ photofixation.

## Graphical abstract

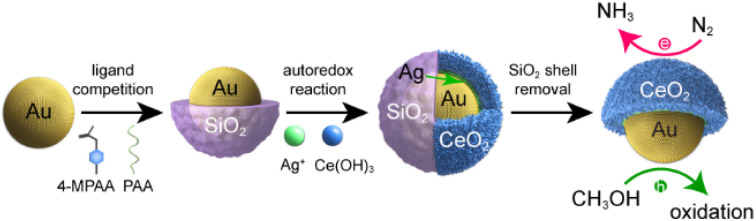

## Introduction

Artificial N_2_ fixation stands out as a potential strategy to convert naturally abundant N_2_ to NH_3_, since NH_3_ is an indispensable compound for all forms of life and modern societies.^1–3^ NH_3_ has recently intrigued interest as an alternative hydrogen carrier owing to its high hydrogen capacity (17.6 wt%) and ease of storage and transportation.^4,5^ Industrially, N_2_ fixation to NH_3_ is achieved *via* the classical Haber–Bosch process with N_2_ and H_2_ as feeding gases, but high temperature (>300 °C) and high pressure (>200 atm) conditions are required to overcome the rate-determining step that is the cleavage of the N≡N triple bond, suffering from its intrinsic energy-intensive nature. Artificial photosynthesis of NH_3_ holds great promise as a flexible alternative to the Haber–Bosch process, because earth-abundant H_2_O other than H_2_ is employed as the reductant and the sustainable solar energy is the only energy source in the N_2_ photofixation process.^6–10^ Among various photocatalysts, semiconductor nanomaterials, especially those contain oxygen vacancies (OVs), have been widely explored for N_2_ photofixation, because the OVs in these catalysts can weaken the N≡N triple bond and activate N_2_ molecules *via* electron donation from the adjacent transition metal complexes, and ultimately promote the reduction of N_2_.^11–19^ An obstacle for the individual semiconductor photocatalyst is the recombination of the generated carriers. To address this issue, the formation of a Schottky barrier at the interface through integrating a semiconductor with a plasmonic metal has proved promising and very useful.^20–25^ Plasmonic metals (*e.g.*, Au nanocrystals) possess unique properties of localized surface plasmon resonance (LSPR), which can not only improve the charge carrier separation but also promote the visible and near-infrared light activity of wide-bandgap semiconductors.^26–28^

However, another challenge arises from the spatial architecture of plasmonic metal/semiconductor photocatalysts.^29–33^ For example, considerable efforts have been devoted to the synthesis of (plasmonic metal)@semiconductor core@shell nanostructures during the past few decades. A disastrous consequence for these symmetric nanostructures lies in that the hot holes generated in plasmonic metals can hardly be accessible by the reactant molecules, because the plasmonic metals are totally buried in the semiconductor shell,^32^ which leads to the recombination of charge carriers. A feasible way to achieve effective charge separation is breaking the symmetry in the growth process to construct asymmetric metal/semiconductor nanostructures, such as dumbbell-shaped or Janus nanostructures.^34–41^ We have previously reported dumbbell-shaped Au/end-CeO_2_ nanostructures through the site-selective growth of CeO_2_ on the ends of Au nanorods.^37^ In contrast to the selective growth on the ends of Au nanorods by virtue of the curvature difference, the selective growth of a semiconductor on the partial surface of Au NSs is also a grant challenge due to the spherically symmetric structures.

In this work, we developed a strategy for the construction of Janus Au NS/CeO_2_ nanostructures with the assistance of a SiO_2_ hard template in combination with an autoredox reaction between Ag^+^ and a ceria precursor. A CeO_2_ semi-shell with sufficient OVs is successfully grown on the partial surface of the Au NS core. In addition, the Janus Au NS/CeO_2_ nanostructures offer an additional opportunity for the selective growth of another noble metal on the exposed surface of the Au NSs to obtain ternary Janus noble metal/Au NS/CeO_2_ nanostructures. Moreover, the Janus Au NS/CeO_2_ nanostructures exhibit excellent photocatalytic performance toward N_2_ photofixation under visible-light illumination, benefiting from the spatially separated reaction active sites. The N_2_ photofixation rate of the Janus Au NS/CeO_2_ nanostructures is 5.3 times higher than that of the core@shell counterparts.

## Results and discussion

**Fig. 1:**
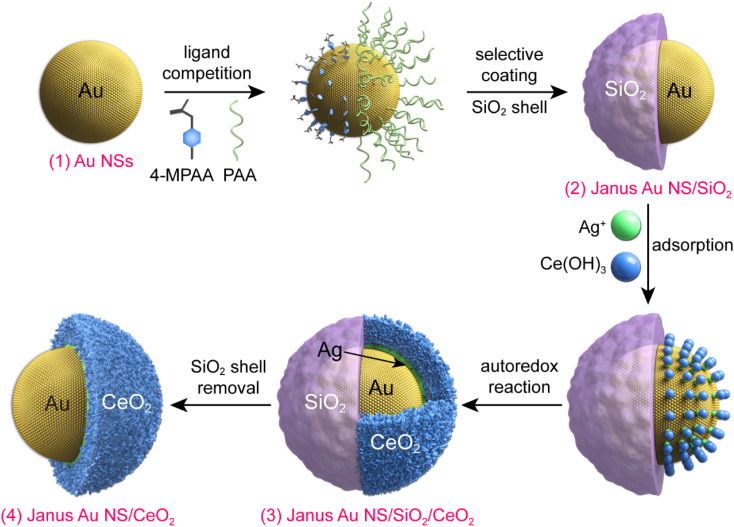
Schematic illustrating the synthesis process of the Janus Au NS/CeO_2_ nanostructures.

Fig. 1 illustrates the synthesis process for the construction of Janus Au NS/CeO_2_ nanostructures. The whole process can be divided into four steps. (1) Growth of the Au NSs: cetyltrimethylammonium bromide (CTAB)-stabilized Au NSs were prepared using a seed-mediated growth method.^42^ (2) Growth of the Janus Au NS/SiO_2_ nanostructures: a SiO_2_ semi-shell is selectively grown on the partial surface of the Au NSs to obtain the Janus Au NS/SiO_2_ nanostructures through competitive ligand coordination between 4-mercaptophenylacetic acid (4-MPAA) and poly(acrylic acid) (PAA).^43^ Specifically, 4-MPAA and PAA ligands self-assemble over Au NSs, resulting in efficient phase separation on the Au NS surface. Since the surface functionalized with the 4-MPAA ligand possesses higher interface energy than the other surface coated with polymer PAA, a SiO_2_ semi-shell preferentially grows on the half surface functionalized with 4-MPAA upon the hydrolysis of the SiO_2_ precursor, leaving the PAA-coated half surface exposed. The SiO_2_ semi-shell is selected as the hard template because the growth of SiO_2_ on Au nanocrystals has been fully exploited.^44–46^ (3) Growth of the ternary Janus Au NS/SiO_2_/CeO_2_ nanostructures: a CeO_2_ semi-shell is therefore selectively grown on the exposed Au NS surface that is functionalized with the PAA ligand *via* an autoredox reaction between Ag^+^ ions and the ceria precursor.^37,47^ Ce(AC)_3_ is employed as the precursor of CeO_2_. It hydrolyzes into Ce(OH)_3_ at a temperature higher than 60 °C. The autoredox reaction is thereafter triggered following the reaction: Ag^+^ + Ce(OH)_3_ = Ag + CeO_2_.^37^ The Ag^+^ ion is selected as the oxidant because the product Ag has a small lattice mismatch with Au (0.2%).^48^ Due to the involvement of a small number of Ag^+^ ions, small Ag crystal nuclei are generated upon the rapid reduction by l-ascorbic acid (AA). These small nuclei can infiltrate the PAA ligand layer and grow on the Au NS surface. In addition, the PAA molecules can function as the stabilizing agent, facilitating the generation of Ag nanocrystals and a CeO_2_ semi-shell. The produced Ag can direct the preferential nucleation of CeO_2_ on the exposed Au NS surface. Once the CeO_2_ nuclei are generated, the ternary Janus Au NS/SiO_2_/CeO_2_ nanostructures are obtained through the overgrowth of the CeO_2_ semi-shell. (4) Etching of the SiO_2_ semi-shell to get the Janus Au NS/CeO_2_ nanostructures: due to the weak alkaline environment and the reaction system temperature (90 °C), the SiO_2_ semi-shell will be gradually etched after the growth of the CeO_2_ semi-shell in about 40 minutes without the addition of an etching agent.

**Fig. 2:**
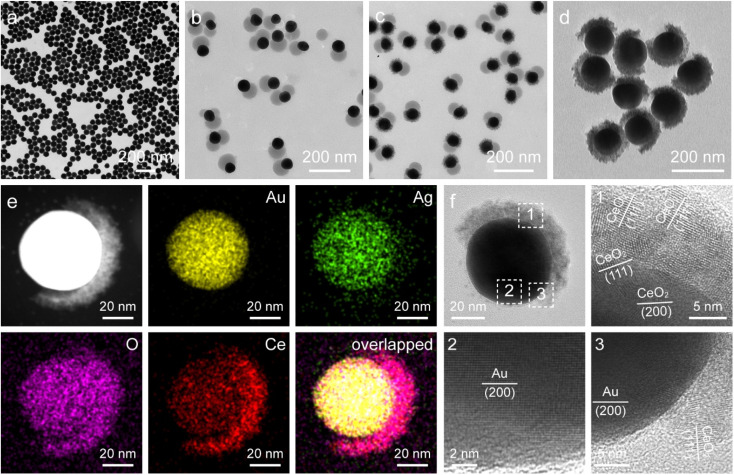
(a–d) TEM images of the Au NSs (a), Janus Au NS/SiO_2_ (b), ternary Janus Au NS/SiO_2_/CeO_2_ (c), and Janus Au NS/CeO_2_ nanostructures (d), respectively. (e) HAADF-STEM image and corresponding elemental maps of a representative Janus Au NS/CeO_2_ nanostructure. (f) HRTEM images of a single Janus Au NS/CeO_2_ nanostructure.

**Fig. 3:**
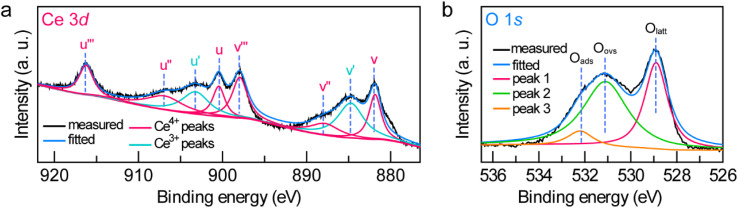
(a and b) High-resolution Ce 3d (a) and O 1s (b) XPS spectra of the Janus Au NS/CeO_2_ nanostructures.

The representative transmission electron microscopy (TEM) images of the Au NSs, Janus Au NS/SiO_2_ nanostructures, ternary Janus Au NS/SiO_2_/CeO_2_ nanostructures, and Janus Au NS/CeO_2_ nanostructures are displayed in Fig. 2a–d. The pre-grown Au NSs possess high shape and size monodispersity, with a diameter of 50.5 ± 2.5 nm (Fig. 2a). After the surface functionalization with two competing ligands, 4-MPAA and PAA, a SiO_2_ semi-shell with a thickness of 39.7 ± 3.6 nm is selectively grown on the partial surface of Au NSs to generate the Janus Au NS/SiO_2_ nanostructures (Fig. 2b). Next, the selective growth of the CeO_2_ semi-shell takes place on the exposed Au NS surface with the assistance of an autoredox reaction and the ternary Janus Au NS/SiO_2_/CeO_2_ nanostructures are obtained in high yield (Fig. 2c). In contrast to the SiO_2_ semi-shell, the CeO_2_ semi-shell looks darker owing to the relatively large atomic number. The successful overgrowth of a CeO_2_ semi-shell on the Janus Au NS/SiO_2_ nanostructures is further confirmed by high-resolution TEM (HRTEM) imaging (Fig. S1†), high-angle annular dark-field scanning transmission electron microscopy (HAADF-STEM) imaging, and energy-dispersive X-ray (EDX) elemental mapping (Fig. S2†), respectively. Intriguingly, the SiO_2_ semi-shell in the ternary Janus Au NS/SiO_2_/CeO_2_ nanostructures is not stable in the reaction system due to the weak alkaline environment and the reaction temperature (90 °C). Eventually, the SiO_2_ semi-shell is etched in about 40 minutes (Fig. S3†). As depicted in Fig. 3d, a CeO_2_ semi-shell with a thickness of 12.3 ± 1.8 nm is selectively grown on the partial surface of the Au NSs to produce the final Janus Au NS/CeO_2_ nanostructures. The thickness of the CeO_2_ semi-shell may be readily tuned in the range of 6.9–21.8 nm by adjusting the amount of ceria precursor (Fig. S4†). The growth process can also be vividly observed from the extinction spectra (Fig. S5†), because the change of the surrounding medium can induce a shift of the resonance peak of Au nanocrystals.^49^ Owing to the significantly larger refractive index of CeO_2_ (2.2) than those of H_2_O (1.33) and SiO_2_ (1.46),^50^ the plasmon resonance peak of Au NSs exhibits a clear redshift (from 525.8 nm to 552.8 nm) after the growth of the CeO_2_ semi-shell, suggesting the formation of the Janus Au NS/CeO_2_ nanostructures.

To gain a better understanding on the structural feature of the Janus Au NS/CeO_2_ nanostructures, HAADF-STEM imaging, EDX elemental mapping, and HRTEM imaging were conducted (Fig. 2e and f and S6†). HAADF-STEM and EDX elemental mapping results clearly demonstrate the Janus feature of the obtained sample (Fig. 2e), consistent with the elemental profile results (Fig. S6†). As displayed in the HRTEM images, the CeO_2_ semi-shell is loosely bound together with small CeO_2_ nanocrystals (Fig. 2f). The formation of crystalline CeO_2_ nanocrystals at a moderate temperature (90 °C) benefits from the autoredox reaction, since a temperature as high as about 400 °C is required to produce crystalline CeO_2_ in traditional synthesis strategies.

To further examine the crystalline nature and chemical states of the Janus Au NS/CeO_2_ nanostructures, X-ray diffraction (XRD) (Fig. S7†) and X-ray photoelectron spectroscopy (XPS) (Fig. 3a and b and S8†) were conducted. The XRD patterns of the Janus Au NS/CeO_2_ nanostructures present two distinct sets of diffraction peaks, one is assigned to the cubic Au phase, and the other belongs to the cubic CeO_2_ phase (Fig. S7†). Ag peaks are absent in the XRD results due to the small amount of metallic Ag in the Janus nanostructures. The presence of elements Au, Ag, Ce, and O in the Janus nanostructures is further ascertained by XPS results (Fig. S8†). The high-resolution Au 4f (Au 4f_7/2_ at 83.3 eV and Au 4f_5/2_ at 87.0 eV) and Ag 3d (Ag 3d_5/2_ at 367.2 eV and Ag 3d_3/2_ at 373.2 eV) XPS spectra suggest metallic Au^0^ and Ag^0^ states in the Janus nanostructures (Fig. S8†).^37,51^ The high-resolution Ce 3d XPS spectrum can be deconvoluted into eight peaks (Fig. 3a). Six peaks belong to the Ce(iv) states, while the other two originate from the Ce(iii) states.^37^ Specifically, there are two series of spin–orbit lines in the Ce 3d XPS spectrum, including Ce 3d_5/2_ (labelled v in Fig. 3a) and Ce 3d_3/2_ (labelled u in Fig. 3a) components.^52,53^ For Ce 3d_5/2_ levels, the v (Ce 3d^9^4f^2^L^*n*−2^, 881.85 eV), v′′ (Ce 3d^9^4f^1^L^*n*−1^, 887.8 eV), and v′′′ (Ce 3d^9^4f^0^L^*n*^, 897.95 eV) peaks belong to the main and satellite characteristic peaks of the Ce(iv) state, while the v′ (Ce 3d^9^4f^1^L^*n*^, 884.74 eV) peak is assigned to the Ce(iii) state.^54^ The Ce 3d_3/2_ levels with the u structures have an identical assignment. The u (900.45 eV), u′′ (906.9 eV), and u′′′ (916.24 eV) peaks are assigned to the Ce(iv) state, while the u′ (903.2 eV) peak is assigned to the Ce(iii) state. The other two peaks (v^0^ and u^0^) corresponding to the Ce 3d^9^4f^2^L^*n*−1^ state are absent in our sample due to tiny distinction with the 3d^9^4f^2^L^*n*−2^ state. The Ce(iii)/Ce(iv) ratio is 0.35/0.65 calculated by integrating the peak areas. The existence of a high concentration of Ce(iii) states (35%) in the CeO_2_ semi-shell corroborates the formation of abundant OVs in CeO_2_ nanocrystals. In addition, the Au/Ce elemental ratio calculated by integrating the XPS peak area is 1 : 2.52. The O 1s spectrum can be fitted with three peaks (Fig. 3b). Peak 1 (528.9 eV) and peak 3 (532.2 eV) represent the crystal lattice oxygen and adsorbed oxygen or hydroxide ions, while peak 2 (531.1 eV) is assigned to the OVs in the CeO_2_ semi-shell,^55^ further confirming the presence of OVs in the as-prepared sample.

**Fig. 4:**
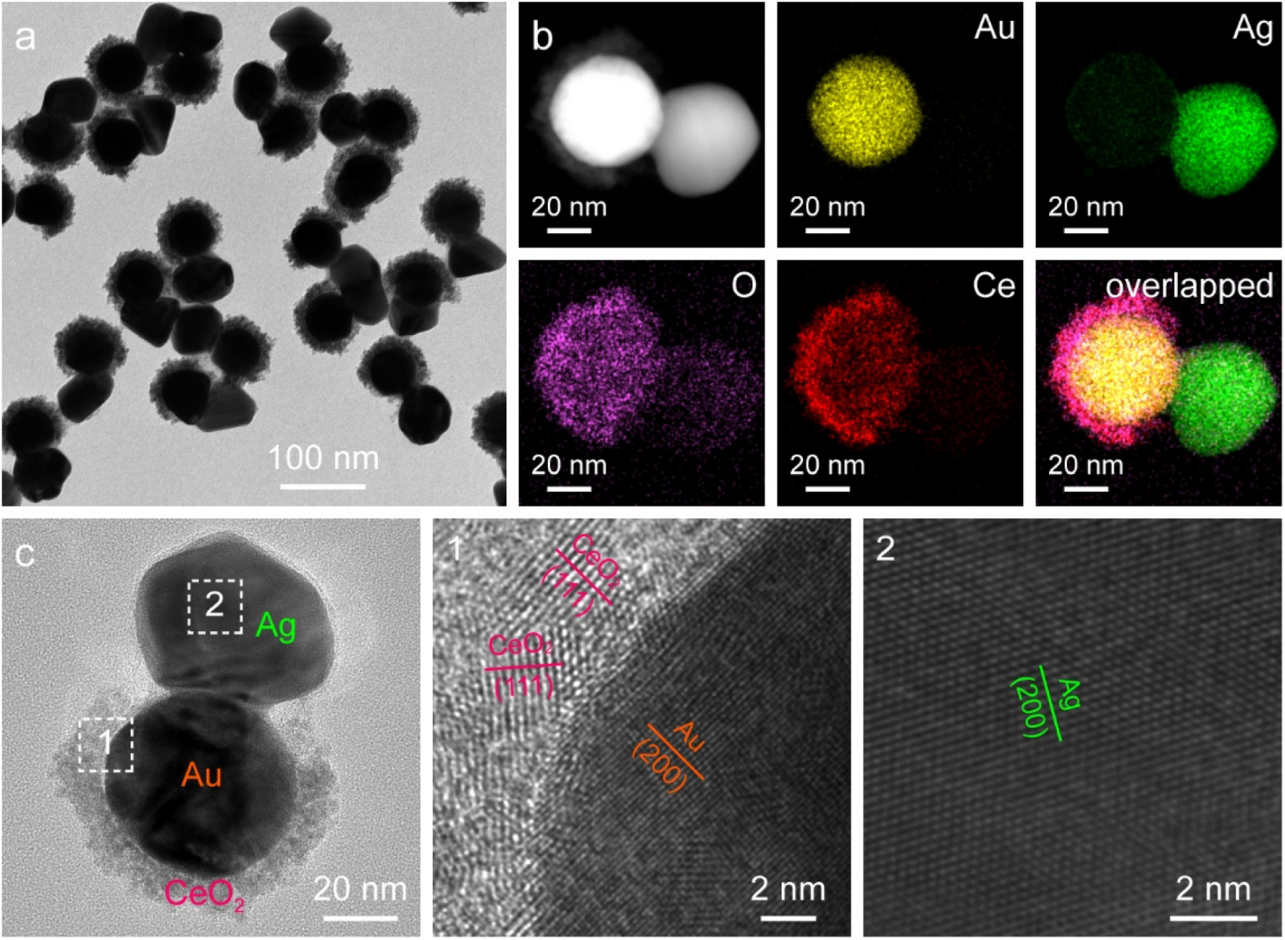
Ternary Janus Ag/Au NS/CeO_2_ nanostructures. (a) TEM image. (b) HAADF-STEM image and corresponding elemental maps. (c) HRTEM images.

The asymmetric Janus Au NS/CeO_2_ nanostructures offer an additional opportunity for the overgrowth of other noble metal nanocrystals (*e.g.*, Ag, Pd, and Pt) on the exposed Au NS surface to produce ternary Janus noble metal/Au NS/CeO_2_ nanostructures. Because the lattice mismatch between the noble metal and Au is much smaller than that between the noble metal and CeO_2_, the noble metal tends to preferentially nucleate on the exposed Au NS surface. As a proof-of-concept, Ag nanocrystals were selected for the overgrowth to fabricate the ternary Janus Ag/Au NS/CeO_2_ nanostructures through the reduction of AgNO_3_ with AA in the presence of polyvinylpyrrolidone (PVP). As displayed in Fig. 4a, Ag nanocrystals with a diameter of about 46.7 ± 4.2 nm are selectively grown on the exposed Au NS surface to obtain the ternary Janus Ag/Au NS/CeO_2_ nanostructures. After the overgrowth of Ag nanocrystals, two new peaks emerge in the extinction spectrum, one is assigned to the plasmon resonance of Ag nanocrystals (426 nm) and the other is originated from the plasmon coupling between Au NSs and Ag nanocrystals (734 nm), suggesting the formation of Ag nanocrystals (Fig. S9†). The successful construction of the ternary Janus Ag/Au NS/CeO_2_ nanostructures is further revealed by the EDX elemental mapping and HRTEM results (Fig. 4b and c). Clearly, element Ag is located on one side of Au NSs, while elements Ce and O are mainly on the other side (Fig. 4b). This is in good agreement with the observation from the elemental profile results (Fig. S10†). In addition, XPS results confirm that the chemical states in the Janus nanostructures remain unchanged after the growth of Ag nanocrystals (Fig. S11†). Similar to the Ag overgrowth, Pd overgrowth on the Janus Au NS/CeO_2_ nanocrystals can be achieved by using cetyltrimethylammonium chloride (CTAC) as the stabilizing agent (Fig. S12†), because CTAC can stabilize the metal ions through the formation of complexes.^45^ The controllable growth of two different types of catalysts (noble metal and CeO_2_) on the plasmonic Au NSs opens a new avenue for exploring diverse photocatalytic applications as bifunctional photocatalysts.

The spatially separated properties of the Janus Au NS/CeO_2_ nanostructures are favourable for charge separation, because the hot electrons and holes can participate in the reduction and oxidation reactions on the CeO_2_ semi-shell and exposed Au NS surface independently. The photocatalytic performance of the Janus Au NS/CeO_2_ nanostructures was evaluated using N_2_ photofixation under visible-light illumination. Methanol was employed as the hole scavenger. For comparison, four other catalysts, Au NSs (Fig. 2a), CeO_2_ nanocrystals (Fig. S13a†), a mixture of Au NSs with CeO_2_ nanocrystals, and Au NS@CeO_2_ core@shell nanostructures (Fig. S13b†), were also prepared. The produced NH_3_ amount was determined using the indophenol-blue method.^24^ A linear calibration relationship was obtained before the photocatalytic experiments (Fig. S14†).

**Fig. 5:**
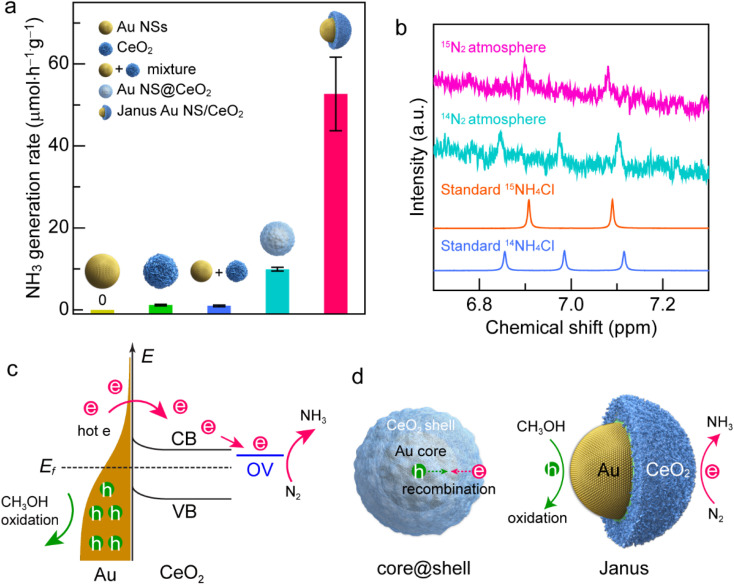
N_2_ photofixation under visible-light illumination. (a) Comparison of the NH_3_ generation rates on the different catalysts. (b) ^1^H NMR spectra of the reaction solution after a 2 h photocatalytic N_2_ reduction reaction using the Janus Au NS/CeO_2_ nanostructures as the catalyst in ^14^N_2_ and ^15^N_2_ atmospheres, respectively. (c) Photocatalytic N_2_ fixation mechanism on the Au NS/CeO_2_ nanostructures. CB, conduction band; VB, valence band; OV, oxygen vacancy states; *E*_f_, Fermi level; e, hot electrons; h, hot holes. (d) Schematics illustrating the hot carrier separation behaviors of the core@shell nanostructure (left) and the Janus Au NS/CeO_2_ nanostructure (right).

Under visible-light illumination, Au NSs and CeO_2_ nanocrystals exhibit very weak N_2_ photofixation activity due to the lack of catalytic active sites or the weak light-harvesting capability (Fig. 5a). The catalytic activity is weak for simply mixing the Au NSs with CeO_2_ nanocrystals, suggesting that the direct contact of Au with CeO_2_ is of great importance to their synergistic effect. In the Au NS/CeO_2_ nanostructures, a Schottky barrier is formed at the interface that facilitates charge separation. The Au NS@CeO_2_ core@shell nanostructures display a slight improvement in the catalytic activity. In contrast, a significant improvement in the photocatalytic activity is obtained for the Janus Au NS/CeO_2_ nanostructures. The generation rate of NH_3_ on the Janus Au NS/CeO_2_ nanostructures is 52.7 ± 9.0 μmol h^−1^ g^−1^, which is 5.3 times higher than that of the core@shell counterparts (Fig. 5a). We compared the photocatalytic activity of the binary (Janus Au NS/CeO_2_) with that of the ternary (Janus Ag/Au NS/CeO_2_) Janus nanostructures as well. The binary Janus nanostructures exhibit higher photocatalytic activity toward the N_2_ photofixation (Fig. S15†). The excellent N_2_ photofixation activity benefits from the spatially separated active sites and the plasmonic properties of the Janus nanostructures. To investigate whether the N_2_ photofixation is driven by the LSPR of Au NSs, we calculated the apparent quantum efficiencies (AQE) by conducting photocatalytic experiments on the Janus Au NS/CeO_2_ nanostructures under the illumination of different monochromatic lights. As displayed in the action spectrum (Fig. S16†), the AQE values faithfully follow the LSPR of the Au NSs, with a maximum at about the LSPR wavelength. The above results confirm that the excellent N_2_ photofixation activity of the Janus nanostructures is driven by the LSPR of Au NSs. To trace the source of NH_3_, control experiments were conducted (Fig. S17†). No NH_3_ is detected under these conditions: (a) without catalysts, (b) in the dark, and (c) under an Ar atmosphere (Fig. S17†), implying that the generation of NH_3_ is originated from the N_2_ photofixation on the Janus nanostructures. The NH_3_ production rate is 9.0 ± 1.3 μmol h^−1^ g^−1^ in the absence of a sacrificial agent, which is much smaller than that obtained in the presence of a sacrificial agent, suggesting that the presence of a sacrificial agent facilitates charge separation and promotes the N_2_ photofixation activity. To further verify the nitrogen source, we performed ^15^N_2_ isotope labelling experiments. As displayed in Fig. 5b, N_2_ photofixation experiments are conducted under ^14^N_2_ and ^15^N_2_ atmospheres, respectively. When ^14^N_2_ is used as the feeding gas, three peaks can be detected in the ^1^H NMR spectrum of the reaction solution, which coincides with the standard ^14^NH_4_^+^ sample. In contrast, when ^15^N_2_ is employed as the feeding gas, two peaks associated with the standard ^15^NH_4_^+^ appear in the ^1^H NMR spectrum. The isotope labelling experiments reveal that the produced NH_3_ in the photocatalytic process is derived from the N_2_ reduction rather than contamination or other sources.^56^ To examine the stability of the Janus nanostructures, TEM imaging and XPS were performed after the typical photocatalytic process (Fig. S18 and S19†). TEM results suggest no morphological changes or aggregation after the photocatalytic process (Fig. S18†). In addition, the chemical states remain unchanged in the Janus nanostructures, as revealed by the XPS results. Moreover, the ratio of Ce(iii) to Ce(iv) is about 0.34/0.66 and is close to that of the as-prepared sample, implying the stability of the OVs on the CeO_2_ semi-shell. To examine the recyclability, we compared the photocatalytic activities of the Janus nanostructures in three successive cycles (Fig. S20†). The Janus nanostructures exhibit excellent recyclability in the N_2_ photofixation. The above results confirm the excellent stability and recyclability of the Janus nanostructures during the photocatalytic process.

The excellent N_2_ photofixation activity of the Janus Au NS/CeO_2_ nanostructures is attributed to their unique architecture and spatially separated active sites. To investigate the activation sites for N_2_ fixation, we performed N_2_ temperature-programmed desorption (TPD) analysis (Fig. S21†). The Janus Au NS/CeO_2_ sample exhibits two peaks. The peak at a relatively low temperature is attributed to N_2_ physisorption, and the strong peak at a higher temperature is originated from N_2_ chemisorption. The presence of the chemisorption peak reveals that the activation sites for N_2_ fixation are the OV sites on the CeO_2_ nanocrystals. In regard to the plasmonic metal/semiconductor photocatalyst, it is commonly known that the Schottky barrier can efficiently promote the charge carrier separation.^20^ Under plasmon excitation, Au nanocrystals harvest light and produce hot electrons and hot holes (Fig. 5c). The hot electrons with sufficient energy can cross the Schottky barrier and inject into the CB of CeO_2_, leaving hot holes in the Au nanocrystals.^37^ Hot electrons and hot holes take part in the reduction and oxidation reactions on the active sites of CeO_2_ and Au nanocrystals, respectively. Since OVs are extensively present on the CeO_2_ surface, a localized electronic state (OV state) is formed and lies below the CB of CeO_2_. Hot electrons are prone to be trapped by the OV-induced defect states. In addition, N_2_ molecules tend to be adsorbed and activated at OV states, followed by reduction with hot electrons.^24^ Meanwhile, hot holes are consumed on Au nanocrystals to close the photocatalytic cycle. Nevertheless, electron–hole recombination occurs if the hot carriers are not consumed quickly.^37,40^ Because the surface of the Au NSs in core@shell nanostructures is totally covered by the CeO_2_ shell, the hot holes left in Au NSs can hardly be accessible and consumed by the hole scavengers, resulting in the recombination of the hot carriers (Fig. 5d, left). In contrast, the active sites for both oxidation and reduction reactions on the Janus Au NS/CeO_2_ nanostructures are fully exposed. N_2_ molecules are reduced by hot electrons on the OVs of the CeO_2_ semi-shell, while hole scavengers (CH_3_OH) are oxidized by hot holes on Au NSs, achieving efficient electron–hole separation (Fig. 5d, right).

## Conclusions

In summary, we have demonstrated a synthetic strategy to construct an asymmetric metal/semiconductor plasmonic photocatalyst for visible-light N_2_ photofixation. With the assistant of a SiO_2_ hard template and an autoredox reaction between Ag^+^ ions and a CeO_2_ precursor, Janus Au NS/CeO_2_ nanostructures featuring spatially separated active sites are successfully obtained. The unique asymmetric nanostructures allow the hot electrons and hot holes to participate in the photocatalytic reactions at the CeO_2_ semi-shell and Au NS surface independently. In addition, the exposed Au NS surface in the Janus nanostructures offers more room for the overgrowth of another noble metal to fabricate ternary Janus noble metal/Au NS/CeO_2_ nanostructures as bifunctional photocatalysts. Moreover, the Janus Au NS/CeO_2_ nanostructures exhibit excellent N_2_ photofixation performance under visible-light illumination. The superior N_2_ photofixation activity benefits from the unique design and spatially separated active sites of the catalyst. This work offers a new strategy for rational arrangement of the active site spatial distribution in plasmonic photocatalysts, shedding new light on the design and construction of spatially separated nanostructures for photocatalytic applications.

## Author contributions

H. L. Jia, M. X. Zhao and A. X. Du contributed equally to this work. H. L. Jia and C.-y. Zhang designed and supervised the project. M. X. Zhao and A. X. Du prepared the materials and performed the photocatalytic tests. Y. R. Dou carried out the product detection. H. L. Jia and C.-y. Zhang wrote the paper. All authors revised the manuscript and discussed the results.

## Conflicts of interest

There are no conflicts to declare.

## Supplementary Material

SC-013-D2SC03863C-s001
