# Supplementary material for: Symmetry-breaking synthesis of Janus Au/CeO2 nanostructures for visible-light nitrogen photofixation
Source: Chem Sci. 2022 Oct 24;13(44):13060–7. doi: 10.1039/d2sc03863c (PMC9667935; doi:10.1039/d2sc03863c)
Supplement: SC-013-D2SC03863C-s001 [file SC-013-D2SC03863C-s001.pdf]

Electronic Supplementary Material (ESI) for Chemical Science.
This journal is © The Royal Society of Chemistry 2022

Electronic supporting information

Symmetry-Breaking Synthesis of Janus Au/CeO_2 Nanostructures for Visible-Light Nitrogen Photofixation

Henglei Jia,[a,‡] Mengxuan Zhao,[a,‡] Aoxuan Du,[a,‡] Yanrong Dou[a] and Chun-yang Zhang*[a]

[a]College of Chemistry, Chemical Engineering and Materials Science, Shandong Normal University, Jinan 250014, China

* Corresponding author. E-mail: cyzhang@sdnu.edu.cn

‡ These authors contributed equally to this work.

Supporting Experimental Section

Chemicals. Sodium borohydride ($NaBH_4$, 99%), silver nitrate ($AgNO_3$, ≥ 99.0%), L-ascorbic acid (AA, ≥ 99%), 4-mercaptophenylacetic acid (4-MPAA, 97%), tetraethyl orthosilicate (TEOS, 98%), cerium (III) acetate hydrate ($Ce(AC)_3 \cdot xH_2O$, 99.9%) were purchased from Sigma-Aldrich. Tetrachloroauric (III) acid tetrahydrate ($HAuCl_4 \cdot 4H_2O$), ammonia solution ($NH_3 \cdot H_2O$, ~25.0–28.0 wt%), hydrochloric acid (HCl, ~36.0–38.0 wt%), isopropanol (≥ 99.7%), and sodium hydroxide (NaOH, ≥ 96.0%) were obtained from Sinopharm Chemical Reagent. Hexadecyltrimethylammonium chloride (CTAC, 97%), salicylic acid ($C_6H_4(OH)COOH$, ≥ 99.0%) and sodium hypochlorite solution (NaClO, available chlorine ≥ 5.0%) were obtained from Aladdin Reagent. Cetyltrimethylammonium bromide (CTAB, > 98%) was purchased from Alfa Aesar. Sodium nitroferricyanide (III) dihydrate ($Na_2[Fe(CN)_5NO] \cdot 2H_2O$) was purchased from Macklin. Poly (acrylic acid) (PAA, MW = 6200, M_w/M_n = 1.09) was purchased from Polymer Source INC. Dimethyl sulfoxide-d6 (DMSO-d6, (D, 99.9%) + 0.03% V/V TMS) was obtained from Cambridge Isotope Laboratories, Inc. Nitrogen ($^{14}N_2$, 99.999%), nitrogen ($^{15}N_2$, 98 atom% ^{15}N), and argon (Ar, 99.999%) were use as received. Deionized (DI) water with a resistivity of 18.2 MΩ·cm was used in all experiments.

Growth of the Au NSs. The monodisperse Au NSs were prepared using a seeded growth method as described in previous work.[1] The whole process can be divided into three steps: (1) growth of the small Au NSs; (2) growth of the large Au nanopolyhedrons; and (3) growth of the large Au NSs.

Growth of the small Au NSs: The small Au NSs were prepared using a seeded growth method. Specifically, the seed solution was made by injecting a freshly prepared, ice-cold $NaBH_4$ solution (10 mM, 600 μL) into an mixture solution composed of CTAB (0.1 M, 9.75 mL) and $HAuCl_4$ (10 mM, 250 μL) under vigorous stirring. The resultant seed solution was kept under gentle stirring for 3 h before use. The growth solution was made by the sequential addition of CTAB (0.1 M, 9.75 mL), $HAuCl_4$ (10 mM, 4 mL), and AA (0.1 M, 15 mL) into DI water (190 mL), followed by the injection of the as-prepared seed solution (0.12 mL). The resultant solution was mixed by gently inversion and kept undisturbed overnigh at room temperature. The obtained small Au NSs were concentrated by centrifugation and redispersion into DI water (55 mL) for further use.

Growth of the large Au nanopolyhedrons: The small Au NSs were employed as seeds for the growth of the large Au nanopolyhedrons. Typically, the small Au NSs (4 mL) was first added into a CTAC solution (25 mM, 30 mL), followed by the sequential addition of AA (0.1 M, 0.75 mL) and $HAuCl_4$ (10 mM, 1.5 mL). The resultant solution was placed in an air-bath shaken (45 °C, 160 rpm/min) for 3 h. The as-prepared large Au nanopolyhedron sample was centrifuged and redispersed into CTAB (20 mM, 30 mL) for further use.

Growth of the large Au NSs: The large Au NS sample was obtained by etching the large Au nanopolyhedron sample with $HAuCl_4$. Typically, $HAuCl_4$ solution (10 mM, 0.2 mL) was added into the large Au nanopolyhedron sample (30 mL) and the mixture solution was placed in an air-bath shaker (45 °C, 160 rpm/min) for 2 h. Large Au NSs were obtained through the mild oxidation of the large Au nanopolyhedrons.

Growth of the Janus Au NS/SiO_2 Nanostructures. The Janus Au NS/SiO_2 nanostructures were prepared through competitive ligand coordination as described in previous work.[2] Typically, 4-MPAA (5 mM in C_2H_5OH, 400 μL) and PPA (0.645 mM in DI water, 400 μL) were added in 2-propanol (50 mL) under vigorous stirring. Then the large Au NS solution with optical density (OD) at the plasmon peak pre-adjusted to 2.0 was added dropwise to the mixture solution. After stirring for 30 min, concentrated ammonia solution (30 wt%, 12 mL) was added, followed by the added dropwise of TEOS solution (8.9 mM in DI water, 1.8 mL). The resultant solution was kept stirring for 12 h to obtain the Janus Au NS/SiO_2 nanostructures.

Growth of the Janus Au NS/CeO_2 Nanostructures. The as-prepared Janus Au NS/SiO_2 sample (10 mL) was centrifuged once and redispersed into DI water (1.8 mL). To trigger the autoredox reaction, $AgNO_3$ (0.1 mM, 100 μL) was added and the resultant solution was kept at room temperature for 1 min to allow for the adsorption of Ag^+ on the Au NSs. Then $Ce(AC)_3$ solution (1 mM, 100 μL) was added. The resultant solution was placed in an oven set at 90 °C for 1 h to produce the Janus Au NS/CeO_2 nanostructures.

Growth of the ternary Janus Ag/Au NS/CeO_2 Nanostructures. The as-prepared Janus Au NS/CeO_2 sample was centrifuged once and redispersed in PVP solution (0.1 M in repeating unit, MW = 40000). Then AA (0.1

M, 20 μL) and $AgNO_3$ (10 mM, 20 μL) were added under vigorous stirring. The ternary Janus Ag/Au NS/CeO_2 nanostructures were obtained after 5 min reaction.

Growth of the ternary Janus Pd/Au NS/CeO_2 Nanostructures. The as-prepared Janus Au NS/CeO_2 sample was centrifuged once and redispersed in CTAC solution containing CTAC (150 μL, 25 wt%) and DI water (10 mL). The mixture solution was stirred for 1 min, followed by the addition of H_2PdCl_4 (30 μL, 0.01 M) and AA (15 μL, 0.1 M) solution. The resultant solution was stirred for 1 min, followed by placing in an oven (30 °C) overnight to obtain the ternary Janus Pd/Au NS/CeO_2 nanostructures.

Growth of the Au NS@CeO_2 core@shell nanostructures. Typically, the large Au NSs (OD = 2.0, 5 mL) was centrifuged at 5500 rpm for 10 min and redispersed in DI water (8.95 mL). CTAB (1 mM, 50 μL), $AgNO_3$ (0.1 mM, 0.5 mL), and $Ce(AC)_3$ (10 mM, 0.5 mL) were subsequently added the Au NS solution under gentle shaking. The overall volume was 10 mL with a final CTAB concentration of 5 μM. The resultant solution was placed in an oven set at 90 °C for 1 h to produce the core@shell nanostructures.

Growth of the CeO_2 nanocrystals. The preparation of CeO_2 nanocrystals was similar to that of the core@shell nanostructures except that the Au NS solution was replaced by DI water and the volumes of DI water (9.55 mL), $AgNO_3$ (0.2 mL) and $Ce(AC)_3$ (0.2 mL) were changed to keep the overall volume to 10 mL.

Photocatalytic N_2 fixation under the visible-light irradiation. The photocatalytic N_2 fixation reaction was carried out in a customized reactor (diameter = 3 cm) with three ends. Two side ends were used as inlet and outlet for gas flow, while the middle end was equipped with a quartz window on the top for light illumination. Typically, the photocatalyst (0.7 mg) was dispersed into DI water (8 mL). CH_3OH (2 mL) was added as the hole scavenger. High-purity N_2 was bubbled in the mixture solution at a speed of 20 $mL \cdot min^{-1}$ for 10 min before each photocatalytic reaction. A continuous Xe lamp (CEL-HXF 300 W) equipped with an AM 1.5 G and a 420−780 nm filters was employed as the visible light source. The optical power density was 300

$mW\cdot cm^{-2}$. A circulation cooling system was used to keep the reaction solution temperature at 25 °C. The N_2 photofixation reaction was performed under visible-light illumination for 2 h. N_2 was bubbled in the reaction solution at a speed of 20 $mL\cdot min^{-1}$ for the entire photocatalytic process. The produced NH_3 in the supernatant was determined using indophenol-blue method.[3,4] Each photocatalytic experiment was repeated three times.

The $^{15}N_2$ isotope labelling experiments were conducted in a customized gastight reactor (diameter = 3 cm) with three ends. Two side ends were used as inlet and outlet for gas flow, while the middle end was equipped with a quartz window on the top for light illumination. The two side ends were closed with glass stopcocks during photocatalytic reaction. Before the light illumination, high-purity Ar was firstly bubbled in the reaction solution at a speed of 100 $mL\cdot min^{-1}$ for 30 min, followed by the $^{15}N_2$ at a speed of 20 $mL\cdot min^{-1}$ for 15 min. After 2-h illumination, the reaction solution was centrifuged to remove the catalyst. The supernatant (500 μL) was acidified with HCl (1M, 200 μL), followed by the addition of DMSO-d6 (300 μL) for ^1H NMR measurements.

Characterization. TEM imaging was conducted on an HT7700 electron microscope operated at 100 kV. The sizes and thicknesses were measured from the TEM images, with more than 300 particles being counted. HRTEM, HAADF-STEM imaging and EDX elemental mapping were performed on an FEI Talos F200S microscopy. The extinction spectra were measured with a Hitachi U-3900 ultraviolet/visible/NIR spectrophotometer. XPS spectra were obtained on a Thermo Scientific ESCALAB 250Xi spectrometer. XRD patterns were performed on a Smart Lab Se diffractometer equipped with Cu Kα radiation. Inductively coupled plasma optical emission spectroscopy (ICP-OES) was carrier out on a PerkinElmer Optima 7300 DV system. ^1H NMR spectra were measured on a Bruker Avance II 400 NMR spectrometer.

Supplementary Figures

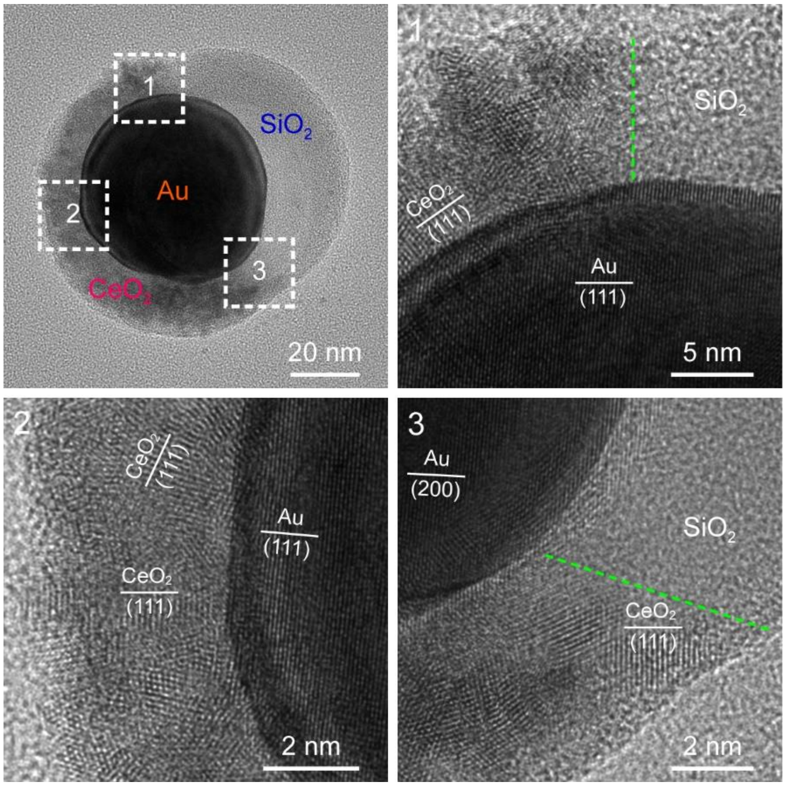

Fig. S1 HRTEM images of a single ternary Janus Au NS/SiO_2/CeO_2 nanostructure and three selected areas.

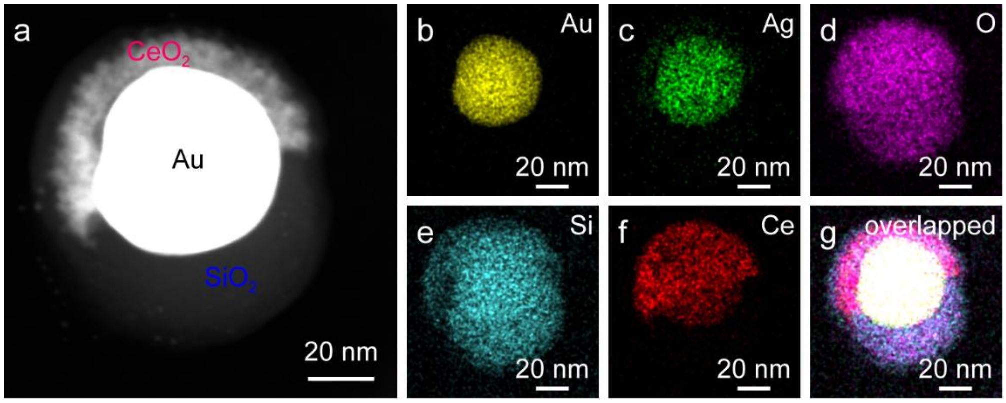

Fig. S2 HAADF-STEM image (a) and corresponding elemental maps (b−g) of a representative ternary Janus Au NS/SiO_2/CeO_2 nanostructure.

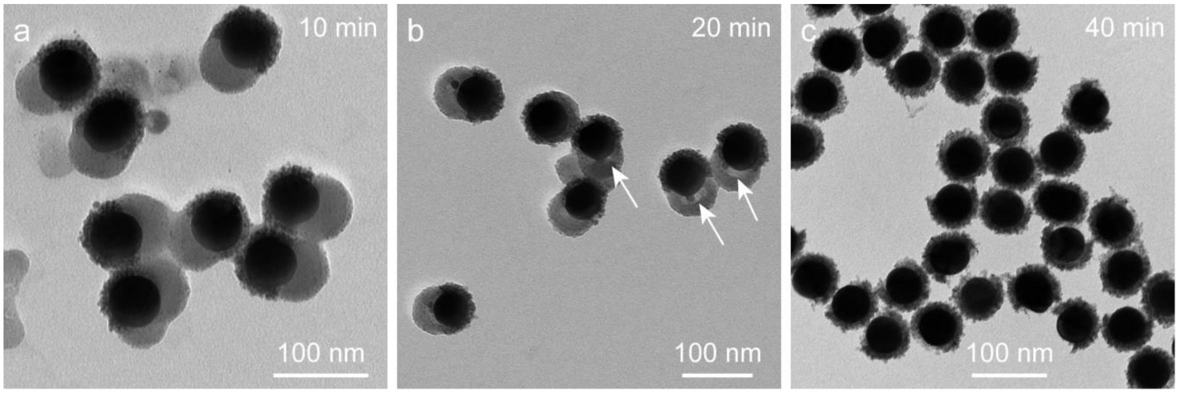

Fig. S3 Representative product TEM images of the Janus Au NS/SiO_2/CeO_2 nanostructures obtained at 10 min (a), 20 min (b), and 40 min (c) of the etching, respectively. The positions marked by white arrows in (b) indicate that the SiO_2 semi-shells are separating from the ternary Janus Au NS/SiO_2/CeO_2 nanostructures.

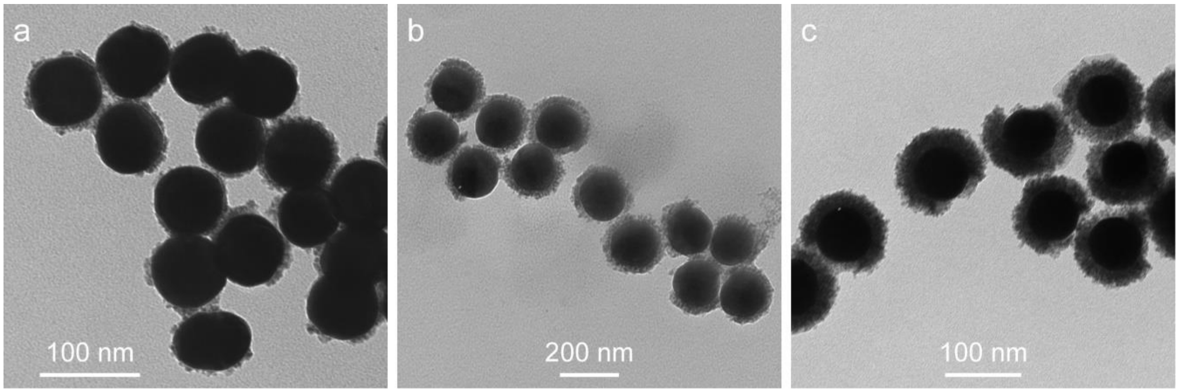

Fig. S4 Effect of the precursor amount upon the CeO_2 semi-shell thickness. TEM images of the Janus Au NS/CeO_2 nanostructures prepared with 50 μL (a), 80 μL (b), and 200 μL (c) $Ce(AC)_3$ solutions, respectively. The thicknesses of the CeO_2 semi-shell are 6.9 ± 1.2 nm (a), 7.4 ± 1.0 nm, and 21.8 ± 2.0 nm (c), respectively.

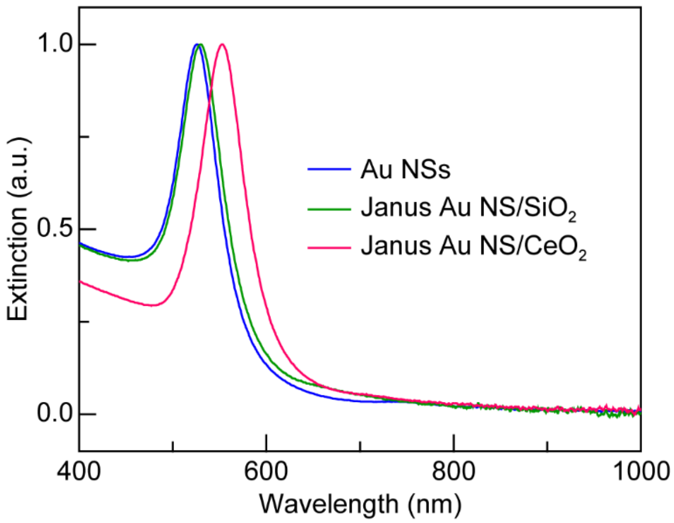

Fig. S5 Extinction spectra of the Au NSs (blue), Janus Au NS/SiO_2 nanostructures (green), and Janus Au NS/CeO_2 nanostructures (red), respectively.

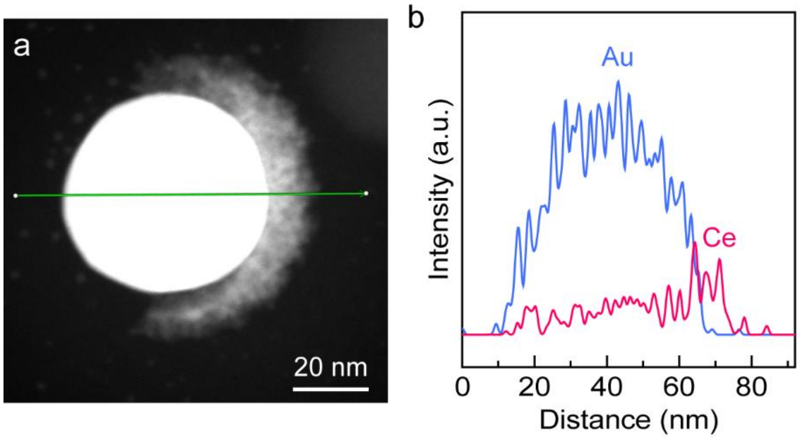

Fig. S6 Elemental profiles of a single Janus Au NS/CeO_2 nanostructure. (a) HAADF-STEM image. (b) Elemental profiles of Au (blue) and Ce (red) acquired along the green line indicated in (a).

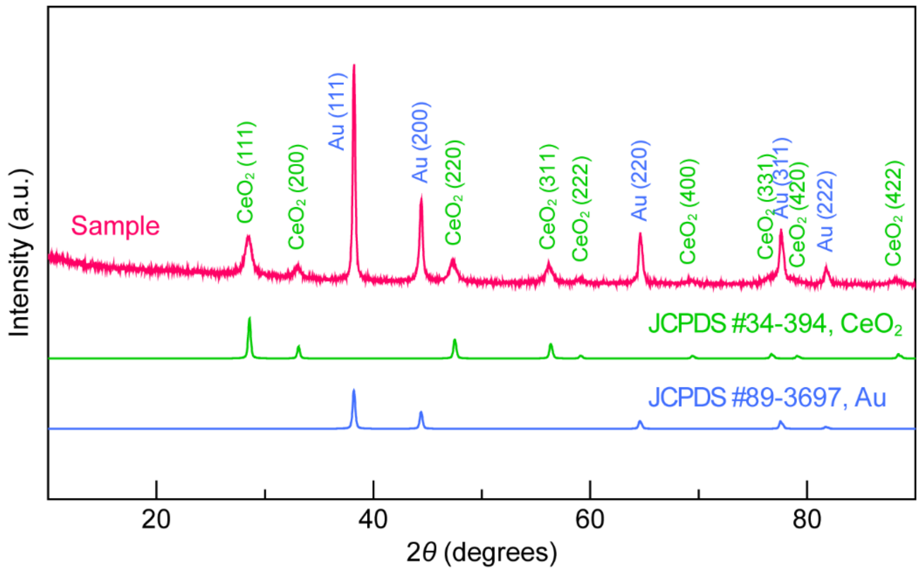

Fig. S7 XRD patterns of the Janus Au NS/CeO_2 nanostructures (red). The blue and green curves are the standard powder diffraction patterns of the face-centered-cubic structure of Au (space group, F*m*-3*m*; lattice constant, 0.40796 nm) and the face-centered-cubic structure of CeO_2 (space group, F*m*-3*m*; lattice constant, 0.541134 nm).

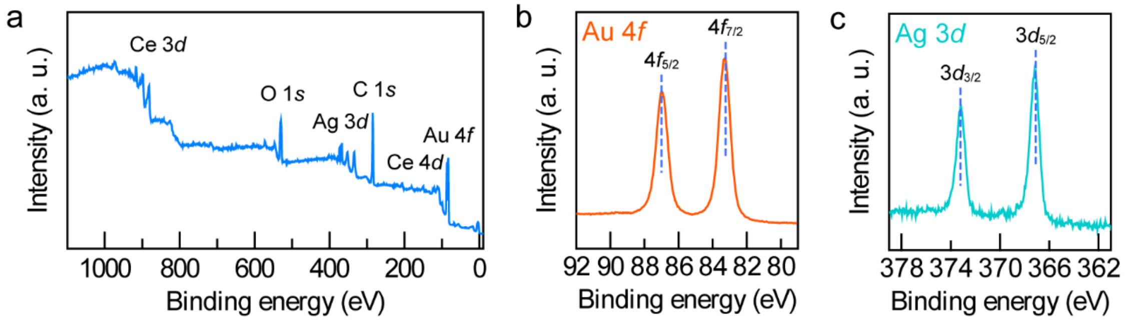

Fig. S8 XPS spectra of the Janus Au NS/CeO_2 nanostructures. (a) Survey spectrum. (b, c) High-resolution Au 4*f* (b) and Ag 3*d* (c) XPS spectra.

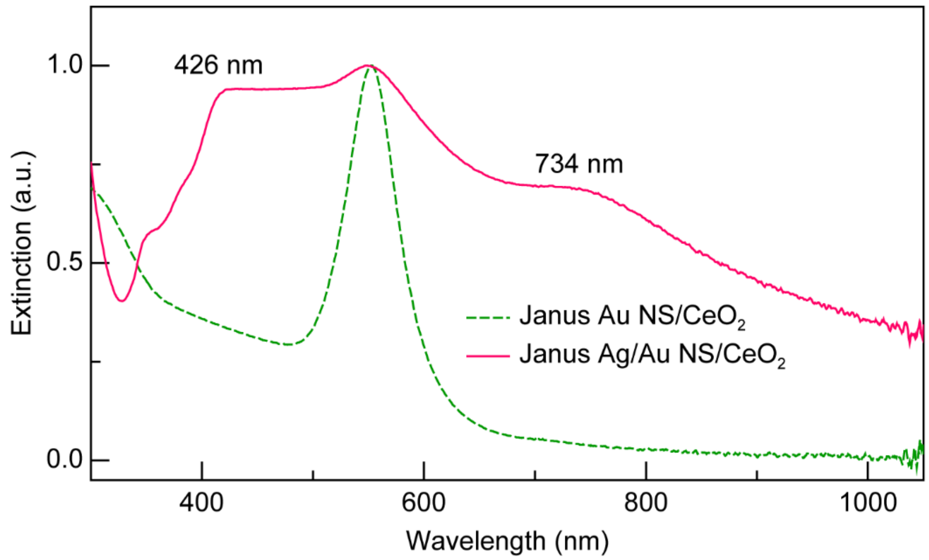

Fig. S9 Extinction spectra of the Janus Au NS/CeO_2 nanostructures before (green) and after (red) the overgrowth of Ag nanocrystals.

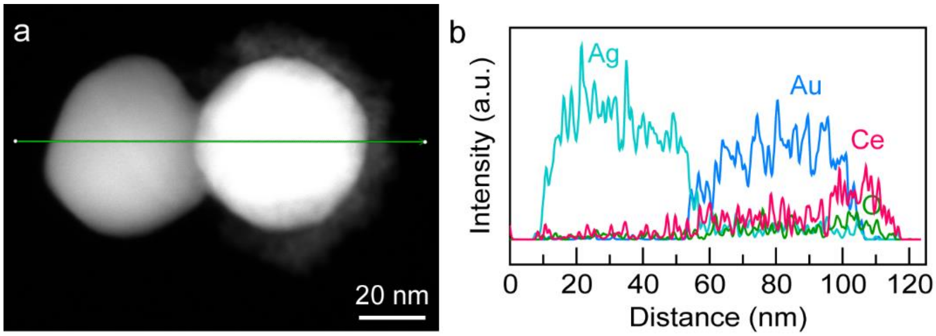

Fig. S10 (a) HAADF-STEM image and (b) corresponding elemental profiles of Au, Ag, O, and Ce acquired along the green line in (a) of a ternary Janus Ag/Au NS/CeO_2 nanostructure.

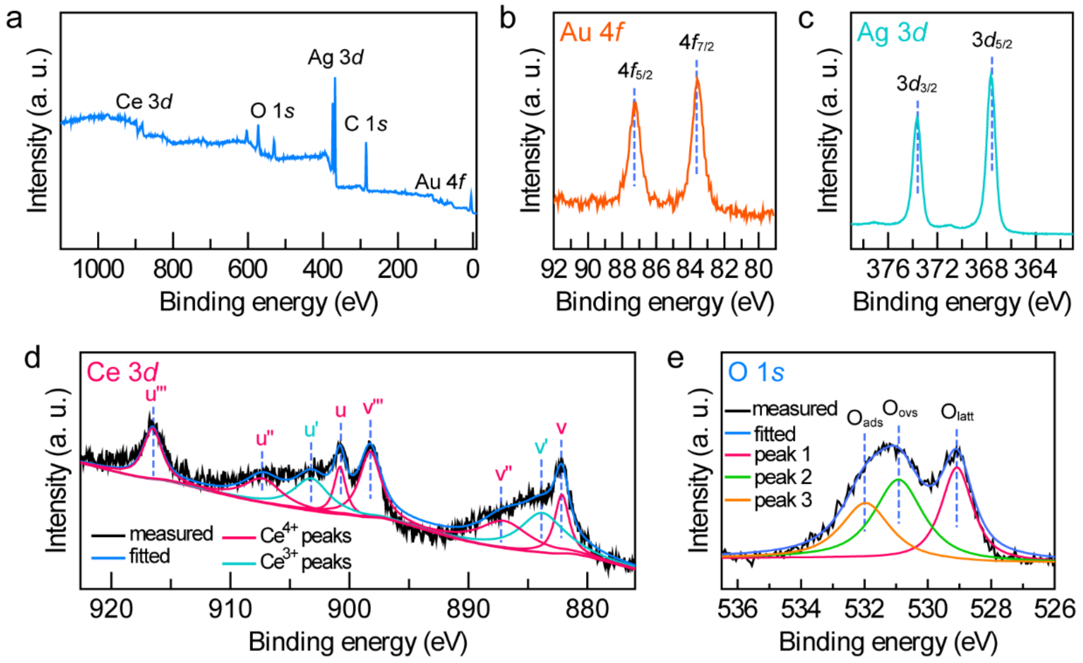

Fig. S11 XPS spectra of the ternary Janus Ag/Au NS/CeO_2 nanostructures. (a) XPS survey spectrum. (b–e) High resolution Au 4*f* (b), Ag 3*d* (c), Ce 3*d* (d), and O 1*s* (e) XPS spectra, respectively.

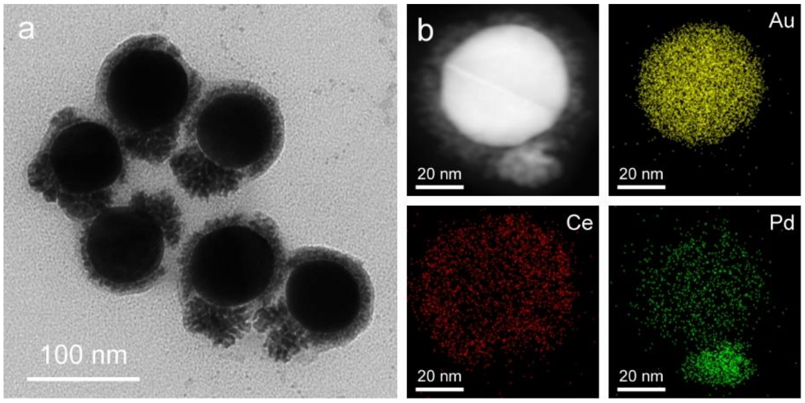

Fig. S12 Ternary Janus Pd/Au NS/CeO_2 nanostructures. (a) TEM image. (b) HAADF-STEM image and the corresponding elemental maps.

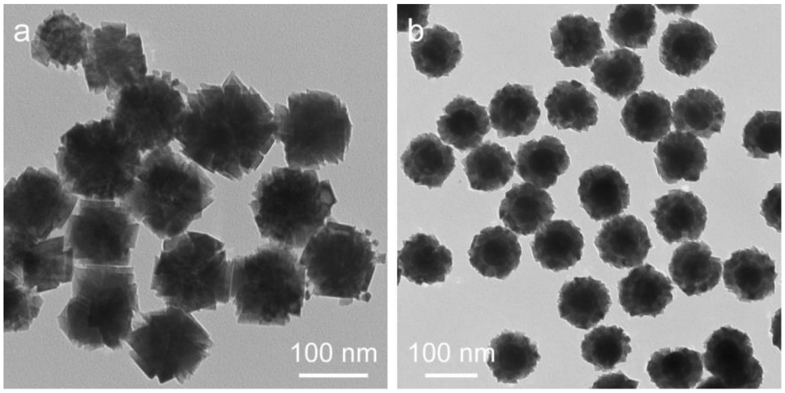

Fig. S13 TEM images of the CeO_2 nanocrystals (a) and the Au NS@CeO_2 core@shell nanostructures (b) used for the N_2 photofixation.

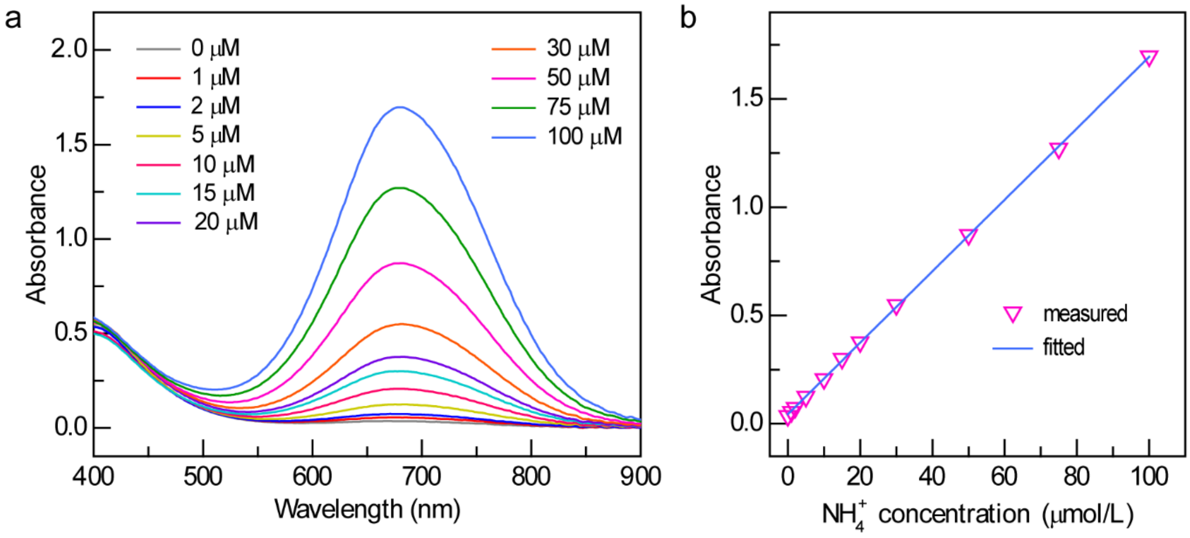

Fig. S14 (a) Absorption spectra of the NH_4Cl solutions with different concentrations. (b) Linear relationship between the absorbance values and the NH_4Cl solution concentrations. The coefficient of determination for the linear fitting is $R^2 = 0.99985$.

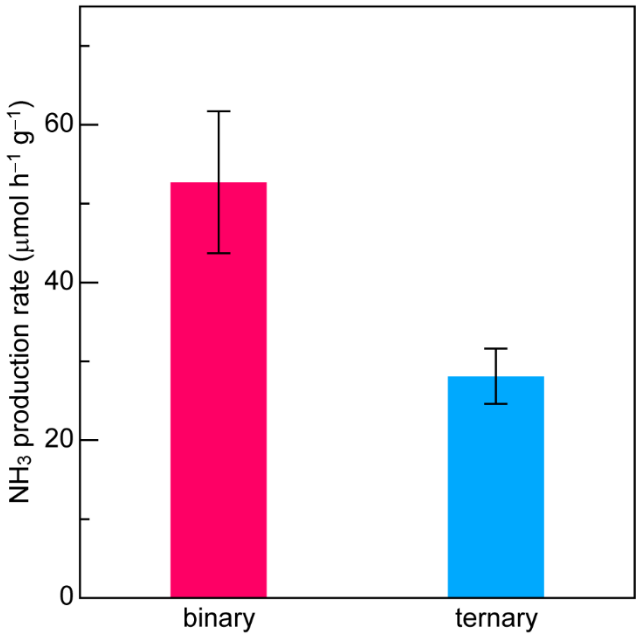

Fig. S15 Comparison of the N_2 photofixation activity of the binary (Janus Au NS/CeO_2) with that of the ternary (Janus Ag/Au NS/CeO_2) Janus nanostructures.

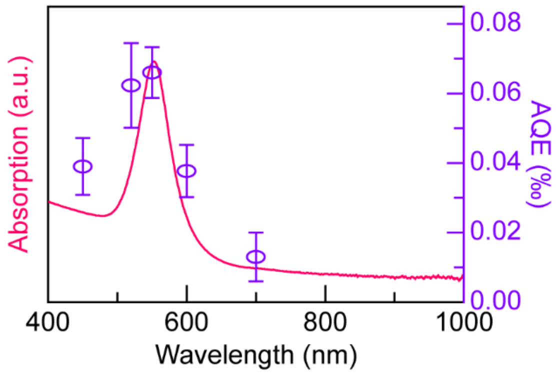

Fig. S16 Extinction (red, left axis) and AQE action (purple, right axis) spectra of the Janus Au NS/CeO_2 nanostructures.

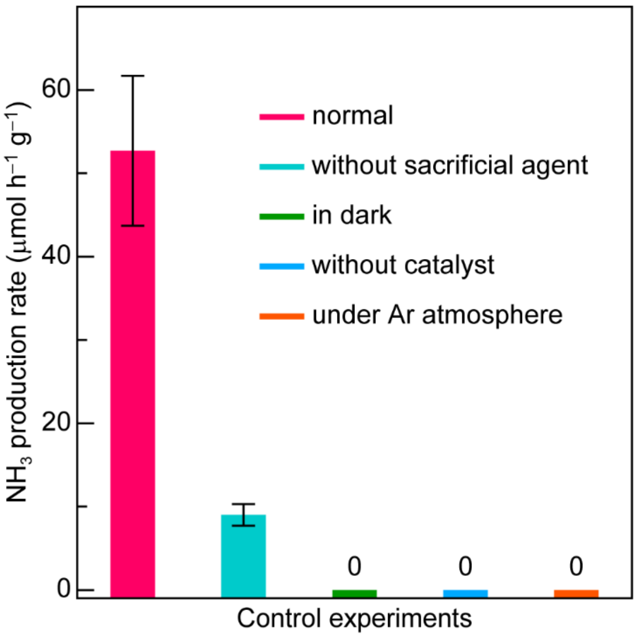

Fig. S17 Control experiments of the N_2 photofixation under different conditions.

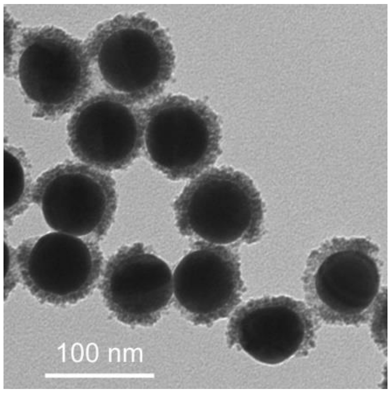

Fig. S18 TEM image of the Janus Au NS/CeO_2 nanostructures after the photocatalytic N_2 fixation.

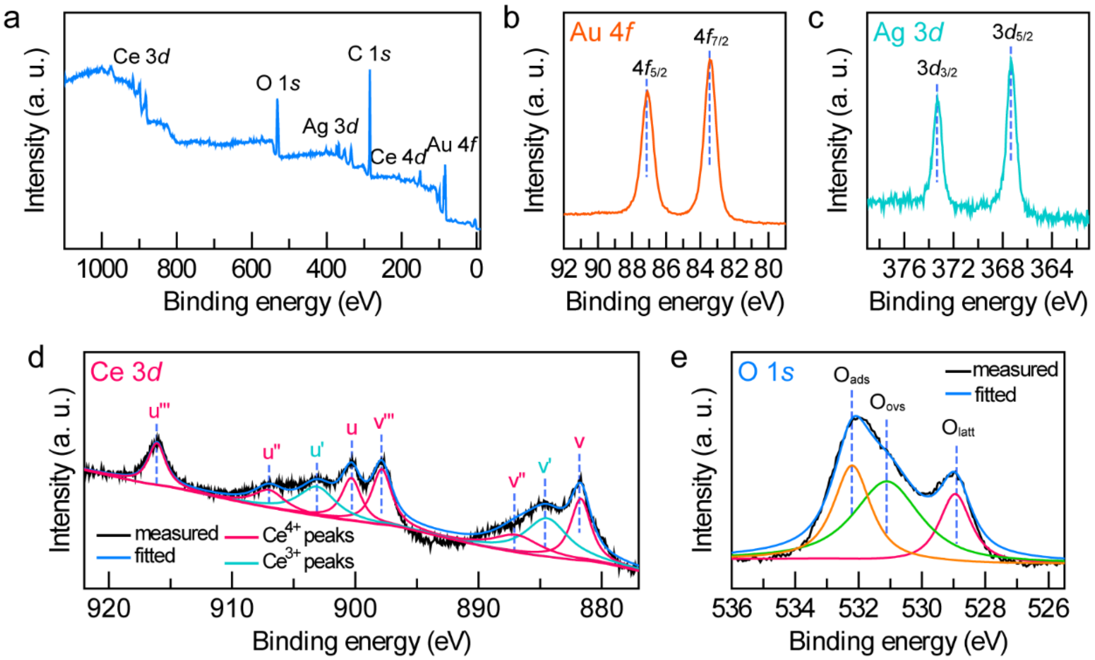

Fig. S19 XPS spectra of the Janus Au NS/CeO_2 nanostructures after the photocatalytic N_2 fixation. (a) XPS survey spectrum. (b–e) High-resolution Au 4*f* (b), Ag 3*d* (c), Ce 3*d* (d), and O 1*s* (e) XPS spectra.

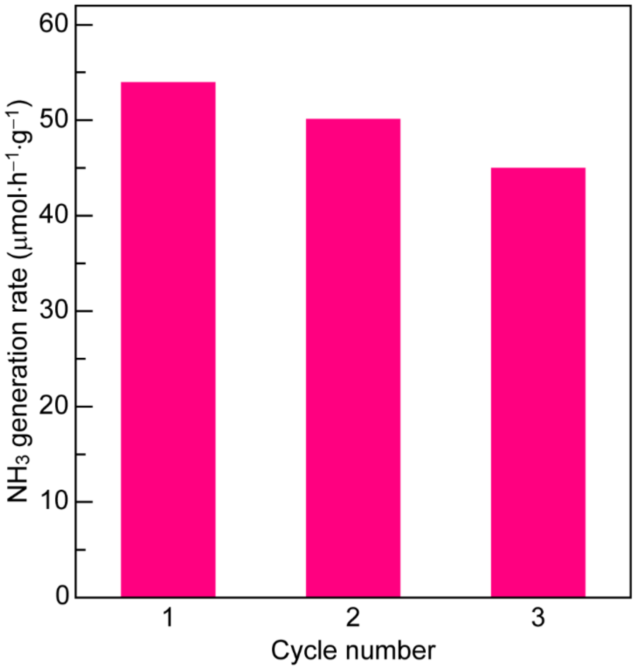

Fig. S20 NH_3 generation rates during three successive cycles with the Janus Au NS/CeO_2 nanostructures as the catalyst.

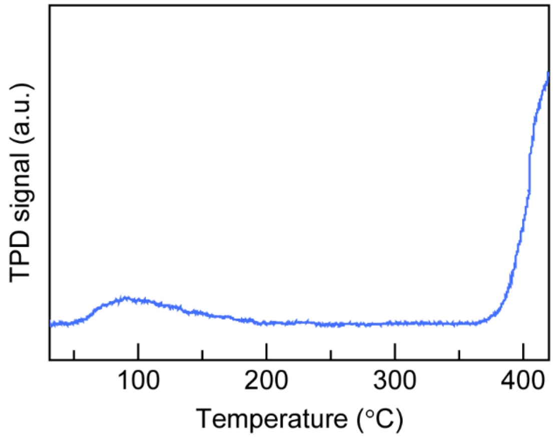

Fig. S21 N_2 TPD profile of the Janus Au NS/CeO_2 nanostructures.

References

1. Q. F. Ruan, L. Shao, Y. W. Shu, J. F. Wang and H. K. Wu, *Adv. Optical Mater.*, 2014, **2**, 65–73.

2. T. Chen, G. Chen, S. X. Xing, T. Wu and H. Y. Chen, *Chem. Mater.*, 2010, **22**, 3826–3828.

3. J. H. Yang, Y. Z. Guo, R. B. Jiang, F. Qin, H. Zhang, W. Z. Lu, J. F. Wang and J. C. Yu, *J. Am. Chem. Soc.*, 2018, **140**, 8497–8508.

4. H. L. Jia, A. X. Du, H. Zhang, J. H. Yang, R. B. Jiang, J. F. Wang and C.-y. Zhang, *J. Am. Chem. Soc.*, 2019, **141**, 5083–5086.
